# Genome Size, rDNA Copy, and qPCR Assays for Symbiodiniaceae

**DOI:** 10.3389/fmicb.2020.00847

**Published:** 2020-05-26

**Authors:** Osama S. Saad, Xin Lin, Tsz Yan Ng, Ling Li, Put Ang, Senjie Lin

**Affiliations:** ^1^State Key Laboratory of Marine Environmental Science, College of Ocean and Earth Sciences, Xiamen University, Xiamen, China; ^2^Department of Biological Oceanography, Red Sea University, Port-Sudan, Sudan; ^3^Marine Science Laboratory, School of Life Sciences, The Chinese University of Hong Kong, Hong Kong, China; ^4^Institute of Space and Earth Information Science, The Chinese University of Hong Kong, Hong Kong, China; ^5^Department of Marine Sciences, University of Connecticut, Groton, CT, United States

**Keywords:** Symbiodiniaceae, genome size, rDNA, Specific-qPCR, HTS, coral

## Abstract

Symbiodiniaceae community structure in corals is crucial for understanding the plasticity of different holobionts under environmental stress. While this relies on molecular analyses, accuracy of molecular quantification, as influenced by DNA extraction efficiency and rDNA copy number variations in particular, has rarely been systematically investigated. Here, we report the development of a set of genus-specific qPCR assays. First, a protocol for efficient DNA isolation and accurate measurements of genome size and rDNA copy number was established. Second, seven newly designed genus-specific ITS2 primer sets were validated using computational and empirical analyses and qPCR assays were developed. We find that while the genome size ranges between 1.75 ± 0.21 and 4.5 ± 0.96 Gbp, rDNA copy number shows over 10-fold variation among Symbiodiniaceae species. Our protocol produced standard curves with high efficiencies (89.8–99.3%; *R*^2^ ≥ 0.999) and tight Cq values over different PCR conditions, illustrating high specificity and sensitivity of the qPCR assays. Tested on mock communities of mixed culture species, our qPCR results agreed well with microscopic counts and facilitated calibration of metabarcoding data. To test the applicability of our protocol for field samples, we analyzed three different Hong Kong coral samples. Six Symbiodiniaceae genera were detected in *Acropora valida, Oulastrea crispata*, and *Platygyra acuta*, with *Breviolum*, *Effrenium, Fugacium*, and *Gerakladium* sp. being reported for the first time. Our results suggest that aggressively disrupting cells to ensure thorough cell lysis, estimating cell loss and DNA loss, and validating qPCR assays are critical for success. The number of species examined here is limited, but the primers are potentially applicable to most species in respective genera, and the protocol and the approach to develop it provide a base and template toward a standardized procedure for quantitatively characterizing Symbiodiniaceae communities in corals.

## Introduction

Symbiotic dinoflagellates in the family Symbiodiniaceae are essential for sustaining coral nutrition and health (Bythell, 1990). Symbiodiniaceae dinoflagellates provide the corals with photosynthetically produced organic carbon and oxygen and are crucial for coral calcification, development and survivability (Stat et al., 2012). Different Symbiodiniaceae species may confer their host corals different levels of fitness and the ability to resist environmental stress and bleaching. Corals are known to each harbor multiple Symbiodiniaceae species (Baker, 2003; Little et al., 2004) and the symbiont community in a coral can shift its dominant species in response to environmental swings (Baker, 2001; Stat et al., 2011). While the mutualistic relationship between Symbiodiniaceae and coral is sensitive to environmental disturbances such as global warming (Kaniewska et al., 2012; Silverstein et al., 2015; Baker et al., 2018), reshuffling of the symbiont communities can increase the host’s resistance, recovery rate and survivability after bleaching events (Berkelmans and Van Oppen, 2006; Lewis et al., 2019). Therefore, information on Symbiodiniaceae community structure in corals is crucial for understanding how the coral-dinoflagellate symbiosis supports coral growth and how it breaks down in events of bleaching. Due to their lack of morphological distinctions, molecular identification and quantification of the dinoflagellate symbionts is essential. In the past decades, an increasing amount of effort has been made to identify and quantify different Symbiodiniaceae species utilizing molecular markers such as 18S, ITS, and 28S domains of the rRNA gene (rDNA). The use of these molecular markers have led to classification of nine clades (Rowan and Powers, 1991; LaJeunesse, 2002; Coffroth and Santos, 2005; Pochon and Gates, 2010). More recently, a systematic molecular analysis combined with morphological comparison has resulted in the formal description of seven of the nine Symbiodiniaceae clades into genera (LaJeunesse et al., 2018), setting a framework to facilitate more taxonomically contextual studies of ecology and evolution of this symbiotic lineage.

The earlier molecular methods such as restriction fragment length polymorphisms (RFLPs) (Rowan and Powers, 1991) temperature gradient gel electrophoresis (TGGE) (Carlos et al., 2000), single stranded conformational polymorphism (SSCP) (Ulstrup and Van Oppen, 2003) had revealed the previously unsuspected genetic differences between Symbiodiniaceae strains. However, these methods largely only detect dominant symbiont types whose contribution was ≥90% among Symbiodiniaceae population and miss background (rare) species (Byler et al., 2013). This was at least partially responsible for the earlier one coral-one Symbiodiniaceae species notion. With the advent of qPCR (Ulstrup and Van Oppen, 2003; Mieog et al., 2007; Correa et al., 2009; Yamashita et al., 2011; Meistertzheim et al., 2019) and DNA metabarcoding (Arif et al., 2014; Quigley et al., 2014; Ziegler et al., 2017) genetic diversity of coral symbionts is no longer disputable. rDNA, particularly ITS, has been well recognized as a powerful marker in documenting Symbiodiniaceae diversity (Ebenezer et al., 2012) due to the presence of variable regions facilitating species resolution and conservative regions enabling universal primer design and their high copy number per genome giving high detection sensitivity (Hillis and Dixon, 1991; Galluzzi et al., 2004; Boulotte et al., 2016; Smith et al., 2017; Hume et al., 2019). However, the prevalent inter-genera or inter-species rDNA copy number variability (LaJeunesse and Thornhill, 2011) hinders accurate translation of metabarcoding data into relative abundances and of qPCR data into their corresponding cell numbers of the constituent species (Ebenezer et al., 2012; Arif et al., 2014; Quigley et al., 2014; Rouzé et al., 2017), all of which is ecologically meaningful information. In face of the problem, some studies have applied a correction factor based on predicted rDNA copy numbers (Mieog et al., 2007; Correa et al., 2009) or genome size data (Lin et al., 2017). However, although the importance of addressing the rDNA copy number variation issue has long been recognized (Thornhill et al., 2007), rDNA copy number variation and its impact on abundance estimation remain understudied for Symbiodiniaceae.

Similarly to metabarcoding, interpretation of qPCR assays using rDNA also suffers from copy number variability (Bustin et al., 2009; Bustin and Huggett, 2017). More fundamentally, the accuracy of qPCR itself also varies with amplification efficiency and DNA efficiency. These potential sources of error have not been systematic examined in development and use of the qPCR and metabarcoding methods. In this work, we established a procedure to establish qPCR assays and developed seven genus-specific qPCR assays, with improvement in ensuring accurate genome size estimation, rDNA copy number estimation, and enhancing the chance of detecting rare species in the Symbiodiniaceae community. We first developed an effective DNA extraction and genome size measurement protocol, which was then used in qPCR assays to establish the ITS copy number for representative species of six Symbiodiniaceae genera and analyze species composition in an artificially mixed community. New primer pairs were designed and evaluated computationally and experimentally, and finally applied to a small set of field coral samples. Our results underscore that using a standardized experimental procedure including a validated protocol, optimized primers, and proper qPCR conditions is important to make data more comparable between studies and more meaningful for ecological interpretations.

## Materials and Methods

### Symbiodiniaceae Cultures

Six Symbiodiniaceae species representing formerly clades A to F (*Symbiodinium microadriaticum*, *Breviolum minutum*, *Cladocopium goreaui*, *Durusdinium trenchii*, *Effrenium voratum*, and *Fugacium kawagutii*) were cultured in f/2 medium (Guillard and Ryther, 1962) at 25°C under a 12:12 light dark regime with a photon flux of 100 μmol m^–2^ s^–1^. Apart from *E. voratum*, which was isolated from East China Sea coastal water, the rest of the dinoflagellates were purchased from the National Center for Marine Algae and Microbiota (NCMA, Bigelow Laboratory for Ocean Sciences, Maine, United States). The growth rate of each culture was checked by daily observation and counting of triplicate samples using a Sedgewick-Rafter counting chamber under a light microscope (Lin et al., 2012). During the exponential growth phase, triplicate samples were collected from each culture using Eppendorf centrifuge 5804R (Eppendorf, Germany) at 4500 × g for 15 min at room temperature (∼20°C). The cell pellets were stored at −80°C until DNA extraction. For the seventh species we attempted to work on, for *Gerakladium* sp. (formerly clade G), we did not have a culture, and instead we used a sample of the natural coral *Oulastrea crispata* collected from Hong Kong, which we found to contain *Gerakladium*, although we could not exclude the possibility that it was a surface contaminant.

### DNA Extraction and Genome Size Estimation With Careful Calibration

DNA was extracted following a previously optimized beat-beating CTAB method (Yuan et al., 2015), including the use of the DNA Clean and Concentrator kit (Zymo Research Kit, Orange, CA, United States). Briefly, samples were incubated at 56°C in DNA extraction buffer for 3 days for cell lysis, and the remaining cells at the end of the incubation were subjected to bead beating on FastPrep-24 bead mill (MP Biomedicals, United States), for repeated cycles of 6 m/s for 1 min, until all cells were broken when a small sample was checked microscopically. Subsequent DNA purification was carried out by removing organic contaminants using Cetyl Trimethyl Ammonium Bromide (CTAB), chloroform, and running the DNA crude extract through DNA Clean and Concentrator kit. The final DNA yield and quality was measured three times on NanoDrop 2000 spectrophotometer (Thermo Fisher Scientific, Franklin, MA, United States) based on absorbance at 260 nm (A_260_) and ratios of A_260_/A_280_ (desired range 1.8–2.0) as well as A_260_/A_230_ (desired range 2.0–2.2). Genome size (pg DNA/cell) of the six cultured species was estimated by dividing the total measured DNA mass by total cell number used for DNA extraction, and the estimation was repeated 10 times for each species. In order to precisely relate the final yield of DNA mass to its corresponding cell number, we included two steps of calibration. Firstly, we counted the cells remaining in the supernatant and in the tube during cell harvesting before it was discarded and subtracted the total cell loss from the original cell number; secondly, we estimated the efficiency of DNA recovery in the DNA purification procedure. This was done after cell lysis by running parallel samples of previously purified DNA samples with known concentrations of DNA through the DNA purification procedure (repeated for 13 times). DNA recovery efficiency was then calculated as (final DNA yield/initial DNA used) ^∗^100. Finally, a linear equation was generated between the initial DNA quantities and the final re-purified DNA quantities for use to calibrate DNA content per cell, which is considered genome size given that the chloroplast and mitochondrial genomes in dinoflagellates are orders of magnitude smaller than the nuclear genomes.

### Amplification of Full-Length ITS and Sequencing

To use in primer design and as standards in qPCR, a 1.5 kbp rDNA region including partial 18S, ITS1, 5.8S, ITS2, and partial 28S (for brevity termed ITS from here on) was amplified from the cultured Symbiodiniaceae species described above using common eukaryotic primer pair; 18ScomF-3end: 5′-GTCGTAACAAGGTTTCCGTAGGTG-3′ and com28SR1: 5′-TCACGCATAGTTCACCATCTTTCG-3′ (Wang et al., 2014). PCR reactions of 50 μl were run in duplicate following the *Ex Tag* PCR kit (Takara Bio, Japan) procedure, under the temperature cycle condition: an initial denaturation for 3 min at 95°C followed by 30 cycles consisting of denaturation at 95°C for 30 s, annealing at 56°C for 30 s, and extension at 72°C for 1.5 min, and a final extension at 72°C for 5 min, on a Bio Red T100 Thermal Cycler (Bio-Rad Inc., United States). However, for *Gerakladium* sp. (formerly clade G) ITS2 fragment of 355 bp was amplified from coral *Oulastrea crispata*, collected from Hong Kong (HK) marginal coral communities (see below), using primer pair ITS-DINO: 5′-GTGAATTGCAGAACTCCGTG-3′ (Pochon et al., 2001) ITS2Rev2: 5′-CCTCCGCTTACTTATATGCTT-3′ (Stat et al., 2009), with the same reaction condition and thermal profile as described earlier, except the annealing and extension steps were 53°C for 30 s and 72°C for 30 s respectively. Amplified fragments were visualized on 1% agarose gel, purified with Clean and Concentrator column (Zymo Research, Orange, CA, United States), and cloned into pMD 19-T vectors (Takara Bio, Japan). Over 20 positive clones for each Symbiodiniaceae species were sequenced using Sanger technology and verified on GenBank Blast search. Those sequences were aligned using ClustalW in MEGA7 software (Kumar et al., 2016), and each species was represented by ITS or ITS2 sequence (*Gerakladium.* sp.) for further experiments.

### Design of Symbiodiniaceae Genus Specific Primers

Existing Symbiodiniaceae species ITS2 database (Cunning et al., 2015, 2017) and about 150 ITS sequences representing the major groups of dinoflagellates and scleractinian coral were retrieved from the NCBI and used along with the ITS representative sequences of our cultured Symbiodiniaceae mentioned earlier to build a custom BLAST database (Supplementary File S1). They were aligned all together in one and genus by genus in another using ClustalW in MEGA7 (Kumar et al., 2016). These all-in alignment and genus-specific alignments were manually inspected and the sequence sites within the rDNA ITS2 region that were specific to each Symbiodiniaceae genus in comparison to other Symbiodiniaceae genera, other dinoflagellates and coral were identified. At least five forward and reverse primer pairs located within each Symbiodiniaceae genus-specific regions were designed using Primer3 online software (Busk, 2014).

### qPCR Assay Optimization and Validation

In order to obtain effective Symbiodiniaceae genus-specific qPCR assays, our newly designed primers were verified and evaluated for primer specificity, sensitivity, efficiency and reliability by applying three different analyses: *in silico* (computational), *in vitro* (cultured samples) and *in situ* (natural coral samples) (Broeders et al., 2014).

#### *In silico* Analysis

Specificity of different primer pair combinations was initially assessed using NCBI BLAST searches^1^. As NCBI BLAST accounts for similarity rather than the thermodynamic stability between the primer and the DNA template, which may weaken the primer specificity test (Qu et al., 2012; Bustin and Huggett, 2017), MFEprimer2 software (Qu et al., 2009) was applied to cover the deficiency in the primer BLAST specificity assessment. MFEprimer2 was run using the ITS database (Supplementary File S1). Primer sets that produced non-specific amplicons were discarded, retaining only primers exclusively targeted their corresponding genus for further verification.

#### *In vitro* Analysis

The nominated primer pairs were experimentally optimized for primer and DNA template concentrations, annealing temperatures, quantification cycle value cut-offs (Cq) and detection limits. Six different cultured Symbiodiniaceae genera and ITS2 PCR product of an uncultured genus (*Gerakladium* sp.) were used in the optimization experiment.

##### Primer optimization

qPCR temperature gradient assays were run for each selected primer pair using three different templates: target (corresponding genus), non-target (other Symbiodiniaceae species) and negative control (MilliQ water). All samples were run using a 12 μl qPCR reaction containing 0.5 μl each forward and reverse primers (10 nM), 6 μl SYBR Green Supermix (Bio-Rad) and 5 μl DNA template equivalent to 1 ng DNA, on Bio-Rad CFX96 Real-Time System (Bio-Rad Inc., United States), under the following thermal condition: an initial denaturation for 3 min at 95°C followed by 36 cycles consisting of 95°C for 10 s, annealing and extension temperature ranging from 54 to 64°C for 30 s, When amplification was complete, melting curve analysis (temperature raised from 65 to 95°C with increment of 0.5°C) was carried out to check whether there was only one kind of amplicon. Primer pairs that successfully returned single kind of amplicon and only generated any amplicon for their corresponding genus excluding all non-target taxa were retained for further verification. Furthermore, for the selected primer pairs (Table 1), one more specificity qPCR assay was run as described above applying 62°C as the annealing temperature, using a duplicate pure DNA sample for each of the cultured Symbiodiniaceae species and purified ITS2 PCR product for *Gerakladium* sp. as respective templates. Finally, PCR products from all target and non-target templates were visualized using 1% agarose gel electrophoresis to verify there was only one band.

**TABLE 1:** The newly designed Symbiodiniaceae genus-specific primers, qPCR assay characteristics and the parameters based on two standard curves: Cell number standard (Cell-STD) and rDNA standard (ITS-STD). Standard deviation of two measurements is denoted as ±.

Target genus	Primer ID	Sequence 5′–3′	Amplicon (bp)	Tm (°C) ^†^	Spec (%)^‡^	Cell-STD E (%)^§^	Cell-STD *R*^2¶^	ITS-STD E (%)^§^	ITS-STD *R*^2¶^
*Symbiodinium*	S.S. ITS2 F	TTCTGCTGCTCTTGTTATCAGG	144	80.0	99.85	97.0 ± 0.99	0.999	97.0 ± 1.56	0.999
	S.S. ITS2 R	ACACACATGAGCTTTTGTTTCG							
*Breviolum*	S.B. ITS2 F	GCAAGCAGCATGTATGTC	126	81.5	99.94	94.8 ± 0.35	0.999	95.3 ± 1.70	0.999
	S.B. ITS2 R	CTTGGAACAACAGTACGCTC							
*Cladocopium*	S.C. ITS2 F	TGCGTTCTTATGAGCTATTGCC	120	79.0	99.98	99.5 ± 0.28	0.999	95.8 ± 0.28	0.999
	S.C. ITS2 R	CAGCGTCACTCAAGTAAAACCA							
*Durusdinium*	S.D. ITS2 F	TTTGCTTCAGTGCTTATTTTACCT	199	83.0	99.95	93.9 ± 0.21	0.999	90.2 ± 1.41	0.999
	S.D. ITS2 R	ACGGCGCAGAAGGACAC							
*Effrenium*	S.E. ITS2 F	GAGGTAAGCTGGACTGATTTG	169	82.0	99.99	98.2 ± 0.49	0.999	93.05 ± 0.92	0.999
	S.E. ITS2 R	TTAGTTCCTTTTCCTCCGCT							
*Fugacium*	S.F. ITS2 F	CCTGTGAGCCATTGAAACTCTAGT	115	78.0	99.96	91.3 ± 3.18	0.999	89.8 ± 0.28	0.999
	S.F. ITS2 R	CAGCGTCACTCAAGAAATACCAT							
*Gerakladium*	S.G. ITS2 F	CAGTGCAATGCCTCCTTGTG	132	83.5	99.99	NA	0.999	94.45 ± 0.92	0.999
	S.G. ITS2 R	CCCACGCATATTCCGGAGA							

*[T_m_ (°C) ^†^]: Melting temperature of the amplicon product. [Spec (%) ^‡^]: Specificity = 1 −Σ [100/2^(Cqi–Cqx^)] (Rouzé et al., 2017), Where: (Cqi) is Cq value obtained from a targeted primer set while (Cqx) from other primer sets using the same DNA sample (data generated using Supplementary Table S2). [E (%)^§^]: Efficiency = [10^(–1/slope^)] – 1. R^2^^¶^: The linear correlation between Cq values and Log (ITS copy No.) or Log (Cell no.). (NA), Not Available.*

##### Standard curves and ITS copy number

For absolute quantification two types of standard curves were established: ITS copy number standard (ITS-STD) and cell number standard (Cell-STD). In ITS-STD, the corresponding plasmid of the representative Symbiodiniaceae genus ITS or ITS2 sequences (see section DNA Extraction and Genome Size Estimation With Careful Calibration) were amplified with M13 vector primers, and the amplicons were purified using Clean and Concentrator column (Zymo Research, Orange, CA, United States). The online OligCalc oligonucleotide properties calculator software^2^ was used to convert the weight concentration of the PCR product into molar concentration, which was then applied in ITS copy number per μl calculation (Hou et al., 2010). Working standard solution was adjusted to 10^9^ ITS copy number μl^–1^, then distributed into 10 μl aliquots for a single use later. Cell-STD was constructed for all Symbiodiniaceae except *Gerakladium* sp. (not cultured) based on cell numbers going into DNA extraction (after calibration for cell loss and DNA loss), and DNA was adjusted to 20 and 40 ng μl^–1^ and distributed into 10 μl aliquots for a single use later. Both standards were stored at -80°C and used within 1 month. Before qPCR runs, the standards were diluted to make 10-fold dilution series, 10^2^–10^7^ copies for ITS-STD and 1, 10, 100, 500, 1000, 2000, and 4000 cell for Cell-STD. Cq values were obtained from each dilution and plotted against log (provided ITS copy number or cell quantity) values for each template concentration. ITS copy number for each species was achieved based on the ITS-STD qPCR results of ITS copy number per unit DNA (IPD) and our estimated DNA quantity per cell (DPC), calculated as IPD x DPC.

##### Analysis of mock community of artificially mixed species

Two sets of mock communities were created by mixing the cultured Symbiodiniaceae species (Supplementary Table S1), one being cell number-based artificially mixed (cMix) and the other being genomic DNA-based artificially mixed (gMix). In cMix, cells from different Symbiodiniaceae species were combined based on microscopic count, and DNA extraction and purification were carried out on the mixture, while in gMix, genomic DNA corresponded to a certain cell number calculated based upon genome size values for each species was mixed with that from other species to generate different mixed samples. These artificially mixed samples along with the standard series and negative control were run in triplicate on qPCR using the same reaction mixture and the thermal profile as defined earlier. For each species, the ITS2 copy number was calculated using the standard curves and transformed to its corresponding Symbiodiniaceae cell number utilizing the ITS copy number data obtained in this study (see section Results). Finally, amplicon of each species was barcoded, pooled with that of other species, and sequenced to verify the species identity of each amplicon. These amplicon sequences were deposited in the NCBI (Accession nos. MN917948- MN917983). Wilcoxon paired two-sided test was run to test the differences between cell number ratios obtained from light microscopy and both qPCR assays. Furthermore, the robustness of the qPCR assays were assessed, which was defined as the consistence of primer over different reaction conditions (Broeders et al., 2014). For each primer set this was tested by constructing a parallel of pure (target genomic DNA) and mixed (target + others genomic DNA) artificial samples at different concentrations and combination ratios. Each pair of samples (pure and mix) was run simultaneously in triplicate following the above-mentioned qPCR assay reactions.

#### *In situ* Analysis

To test the specificity and sensitivity of the qPCR assays as applied to natural coral samples, a small number (three) of scleractinian coral species, *Acropora valida*, *O. crispata*, and *Platygyra acuta* (*n* = 2, 5, and 4 respectively), were selected to represent different natural symbiotic Symbiodiniaceae genera based on HTS results (unpublished data). These three corals were collected from Northeastern HK marginal coral communities and Symbiodiniaceae species diversity had previously been tested using denaturing gel gradient electrophoresis of ITS2 (Ng and Ang, 2016). Those samples were preserved in 85–90% ethanol at -20°C and then dried and smashed before adding the DNA lysis buffer. At the time of analysis samples were thawed at room temperature and homogenized using the same method as for cultured Symbiodiniaceae. DNA isolation, the qPCR assays and sequencing were performed as described above. All qPCR retrieved sequences were deposited in the NCBI (Accession nos. MN917869-MN917947). The absolute cell number quantification was obtained directly by applying the Cell-STD standard curve. However, for *Gerakladium* sp. the absolute quantification was not possible because its genome size and rDNA copy number data were not available. Instead, the equation for relative quantitation, [2^Cq(G)^ ^–Cq(C)^] (Correa et al., 2009), was used to assess its relative cell abundance equivalent to *C. goreaui*, assuming same genome size and rDNA copy number as *Cladocopium*, where Cq (C) are the Cq values of *C. goreaui*.

### Illumina HiSeq Sequencing and Processing Incorporating rDNA Copy Number

Five artificially mixed Symbiodiniaceae DNA (gMix) samples with different species to species ratios (Supplementary Table S1) were prepared. The ITS2 region was amplified using the ITS-DINO and ITS2Rev2 primers containing additional eight bases as barcode sequence specific to each sample. The amplicons were purified, pooled and paired end sequenced (2 × 250) on an Illumina sequencing platform. Reads with > 10% unknown nucleotides or containing <80% of bases >20 (Q-value) were removed. The clean paired end reads with minimum overlap of 10 bp and <2% mismatch were merged using FLSAH (Magoè and Salzberg, 2011) as raw tags, and filtered using QIIME (Caporaso et al., 2010). Primer and barcode sequences were trimmed from the 3′ and 5′ ends and the tags without the primer sequences were discarded. Tags in which the continuous low quality bases > 3 and tags shorter than 300 bp were discarded. The resulting cleaned tags were clustered into operational taxonomic units (OTUs) of 97% similarity with UPARSE pipeline (Edgar, 2013), and then the tag with highest abundance within each OTU was nominated as representative sequence of the OUT. Raw sequence data were deposited in NCBI (SRA) under the accession number PRJNA599937. The representative sequences were assigned into Symbiodiniaceae ITS2 type (sub-genera or strain) following previous definitions (Silverstein et al., 2015; LaJeunesse et al., 2018) by Blastn against the custom Symbiodiniaceae ITS2 type database. The Symbiodiniaceae ITS2 database used for the BLAST analysis was constructed by augmenting existing database (Cunning et al., 2017) with our sequence data from the cultured species (Supplementary File S2). The count of sequences of each ITS2 type within a given sample were transformed into their relative abundance, and ITS2 types accounting for <0.05% of total sequence counts were excluded from the quantitative analysis (Cunning et al., 2017). The relative abundance of each ITS2 type were then calibrated using the rDNA copy number obtained in this study.

### Data Analysis

Statistical analyses and graphics were achieved using GraphPad Prism (version 8.3.0) and *R* (version 3.4.4) software (R Core Team, 2018). Triplicate measurement data were reported as mean ± SD. Prior to difference analysis all data were checked for normality using Shapiro–Wilk test. Pearson correlation coefficient was run to assess the strength of correlations between the final and the initial DNA amount. To evaluate the consistency of the qPCR primers over different reaction conditions, paired two-side t-tests were run between the mix and pure sample sets. Wilcoxon signed-rank tests were performed to examine whether the Symbiodiniaceae proportion differed significantly between microscopic count, results of Cell-STD based qPCR assays and ITS-STD based qPCR assays. Furthermore, Bland-Altman tests were used to assess the agreement between microscopic count and results of both types of qPCR assays. The significant value for all the statistical analyses was set at *p* = 0.05.

## Results

### DNA Recovery Efficiency and Symbiodiniaceae DNA Contents

**FIGURE 1:**
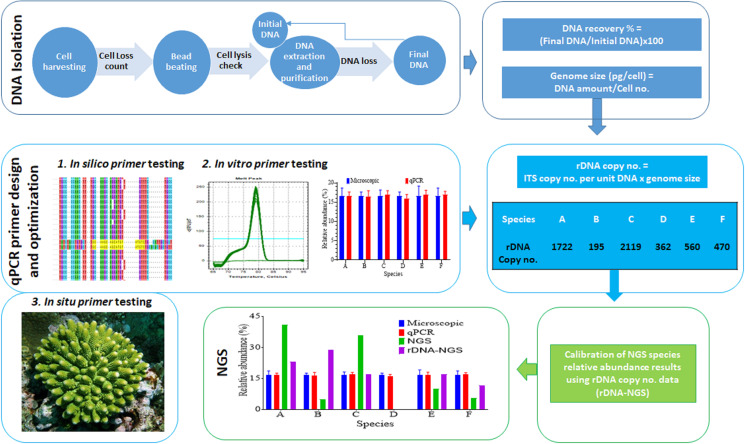
Workflow of the experimental procedure. It mainly consists of DNA recovery rate and DNA content per cell assessment (top row boxes), development of qPCR specific assays (second row boxes), and calibration of NGS data using predicted rDNA copy number per cell (bottom row boxes).

**FIGURE 2:**
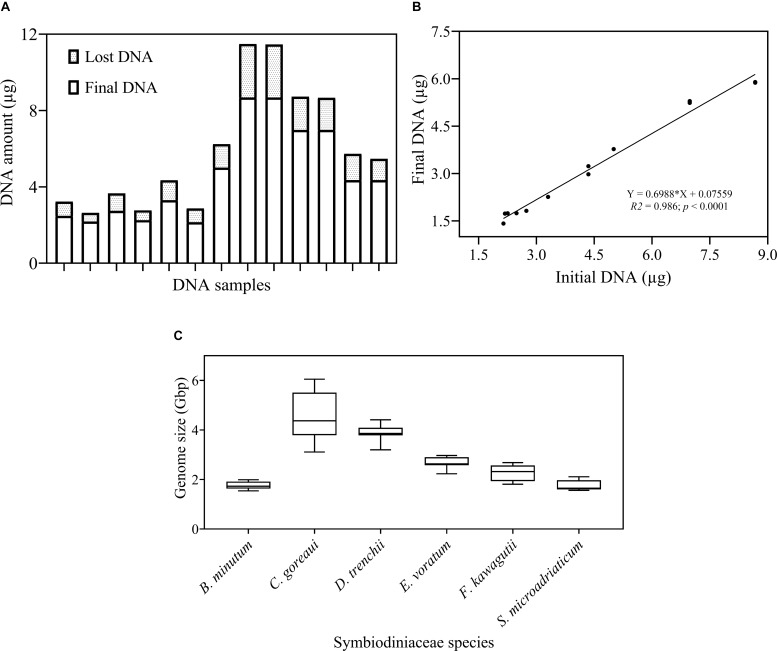
Examination of DNA recovery efficiency and Symbiodiniaceae genome size. **(A)** The proportion of DNA amount (μg) lost during the DNA isolation and purification process. **(B)** Pearson correlation between initial and final DNA concentration. Shown are means of triplicate measurements. **(C)** Genome size (Gbp) of each species estimated as DNA mass per cell after calibrations for cell loss using the linear equation in **(B)**. Box boundaries represent the 75th and 25th percentiles. Thick lines within the boxplots represent the mean. Whisker bars above and below the boxes represent the 95th and 5th percentiles. The conversion factor, 1 pg DNA = 0.978 Gbp (Dolezel and Bartos, 2005) was used in genome size estimation. Each value is an average of triplicates.

The protocol developed in this study consists of three main steps: DNA extraction efficiency and genome size measurement, qPCR primer development and optimization and calibration of HTS data (Figure 1). We first examined the DNA recovery efficiency of our DNA extraction and purification procedure, defined as proportion of the final DNA yield to its original amount provided in the DNA extraction and purification procedure. Results showed that the lost DNA was proportional to original DNA, giving a highly stable DNA recovery efficiency (71.71 ± 4.54%) across different DNA mass (Figure 2A). This was confirmed by the strong positive Pearson correlation coefficient (*r* = 0.993; *p* < 0.0001) between the initial and final DNA masses (Figure 2B). This efficiency was then used to compute the actual final DNA mass in each DNA extraction effort and to estimate Symbiodiniaceae species genome sizes. Among the six species examined in the study, *C. goreaui* exhibited the largest genome, 4.50 ± 0.96 Gbp per cell, while *S. microadriaticum* and *B. minutum* possess the smallest genomes, 1.75 ± 0.21 and 1.76 ± 0.16 Gbp per cell, respectively (Figure 2C).

### Primer Sets Optimization and Validation

The second step of our protocol was to validate primers and optimize qPCR conditions (Figure 1). Seven Symbiodiniaceae genus-specific ITS2 primer pairs (Table 1) were designed and evaluated using computational and experimental assays following the common qPCR guidelines (Bustin et al., 2009; Broeders et al., 2014). All primer sets were verified to be specific to their respective target genera from the results of NCBI primer search and MFEprimer2 analyses (Supplementary File S3). A small number of putative non-target sequences were returned from the primer blast search; however, further blast search against the GenBank database revealed that those sequences had been misannotated (Supplementary Table S3). Results of qPCR assays also showed high specificities for each primer set as none of them amplified DNA templates from non-target cultured Symbiodiniaceae genera (Table 1 and Supplementary Table S2). In these assays, no primer dimer was detected in any reactions and no amplicon was generated in the negative control (Milli Q water), indicative of high quality primers and absence of contamination in the reaction system. Furthermore, all target amplicons displayed a single melting temperature peak (Tm), which was higher than 78°C, demonstrating that only one amplicon sequence was generated (Supplementary Figure S1). Finally, agarose gel electrophoresis of the amplicons showed one single band for target genus with molecular size as expected of the target amplicon and no bands were generated from non-target genera, confirming again the specificity of the primer sets (Supplementary Figure S1).

**FIGURE 3:**
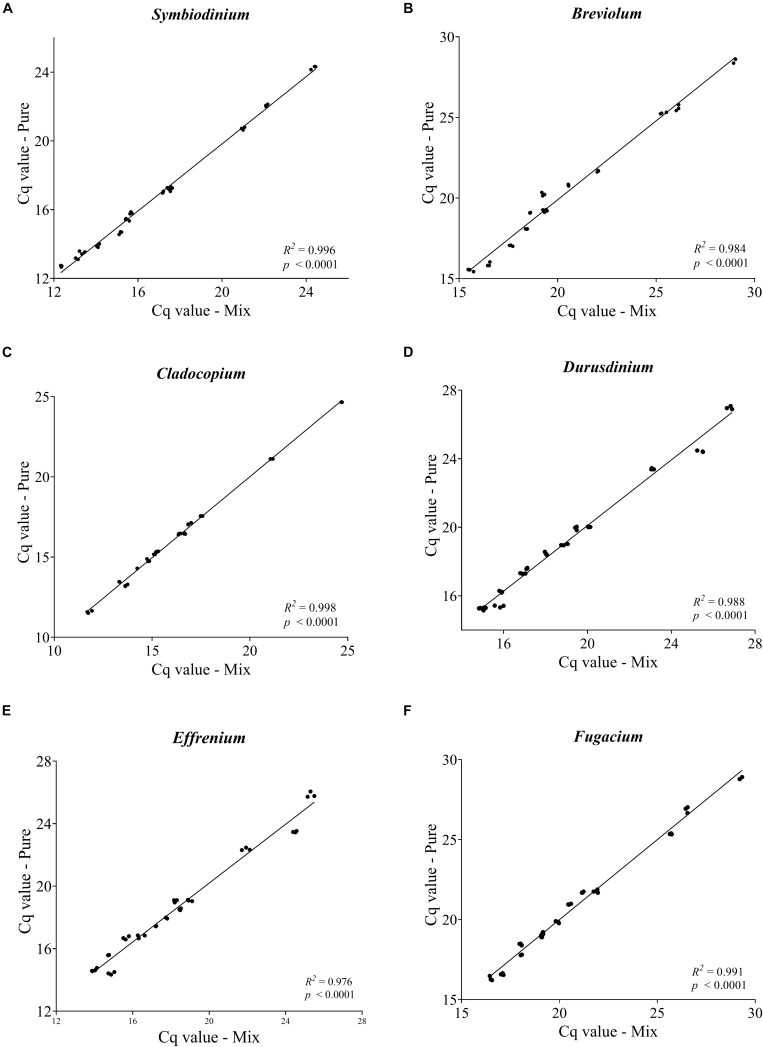
Pairwise comparison of Cq values obtained from each targeted genus within a mixture with non-targeted Symbiodiniaceae genera. Each plot **(A–F)** represents one Symbiodiniaceae genus-specific qPCR primer pairs with genus name shown on the top. Overall variations in Cq values were < 0.5 except for some of the *Effrenium* and *Durusdinium* values which ranged between 0.5 and 1. 04. Each point is an average of triplicates.

As a sign of qPCR assay robustness, the consistency across different samples or reaction conditions was analyzed. High consistency was illustrated from the discrimination between the target genera in a mixture and the comparable abundance estimates for each target genus from the mixed sample and pure species sample. Differences in Cq value between the two types of samples would indicate qPCR biases caused by the co-existence of non-target species. No significant differences were found (*paired two-side t-test; p* > 0.05) between the pure and the mixed target for any of the primer pairs (Figure 3). Furthermore, the robustness of the qPCR assays were also indicated by the stable Cq values when the annealing temperature changed from 56 to 64°C, over which the differences in Cq values were ≤ 0.5 except for *Breviolum* and *Effrenium* assays, in which Δ Cq variations of 1.54 and 0.72 were found, respectively (Supplementary Table S4).

### Standard Curves

Standard curves are critical for absolute qPCR quantitative analyses of samples. Here, we compared two types of standard curves: cell number based standard (Cell-STD) and ITS copy number based standard (ITS-STD). Results showed that each genus-specific primer set exhibited a good linear regression between the Cq value and logarithmic of the starting cell number or starting rDNA ITS copy number. The qPCR assays displayed a detection limit of 10 gene copies, which is less than one cell per reaction due to multiple copies of rDNA in each cell. The standard curves between the Cq value and log [Starting Quantity (SQ)] for all species were parallel to each other and all displayed a strong linear correlation, with a coefficient of correlation *r* > 0.999 (*p* < 0.001) and a coefficient of determination *R*^2^ = 0.999, for both types of standards (Supplementary Figure S2). The efficiency of amplification reactions for all primer sets was also consistent and high over all assays performed for both ITS-STD and Cell-STD, which ranged from 89.8 to 99.5% (Table 1). Furthermore, consistency among different assays, with low Cq value variations between replicates (<0.5), also indicated the reliability of the quantitative results.

### Estimation of Symbiodiniaceae ITS Copy Numbers

**FIGURE 4:**
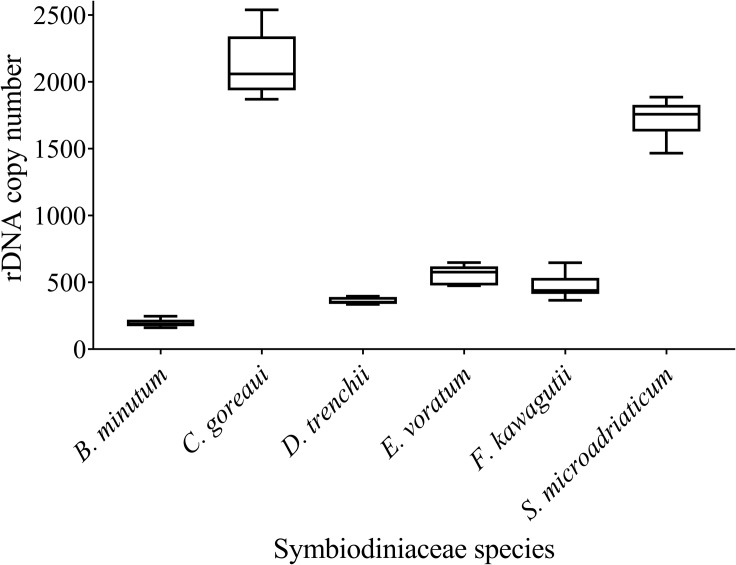
rDNA copy numbers generated for six Symbiodiniaceae species using the Symbiodiniaceae genus-specific qPCR assays based on ITS copy number standard curves. Box boundaries represent the 75th and 25th percentiles. Thick lines within the boxplots represent the mean. Whisker bars above and below the boxes represent the 95th and 5th percentiles.

We found a wide range of ITS copy number for species examined in this study, ranging from the highest 2119 ± 217.17 in *C. goreaui* to the lowest 195 ± 28.97 in *B. minutum* (Figure 4). While *C. goreaui* was the highest in both ITS copy number and genome size, *S. microadriaticum* possessed the second highest ITS copy number yet the smallest genome (Figures 2C, 4). In contrast, *D. trenchii* with relatively high genome size possessed lower rDNA compared to *E*. *voratum* and *F*. *kawagutii* (Figures 2C, 4).

### PCR Quantification of Symbiodiniaceae in Artificially Mixed Samples

**FIGURE 5:**
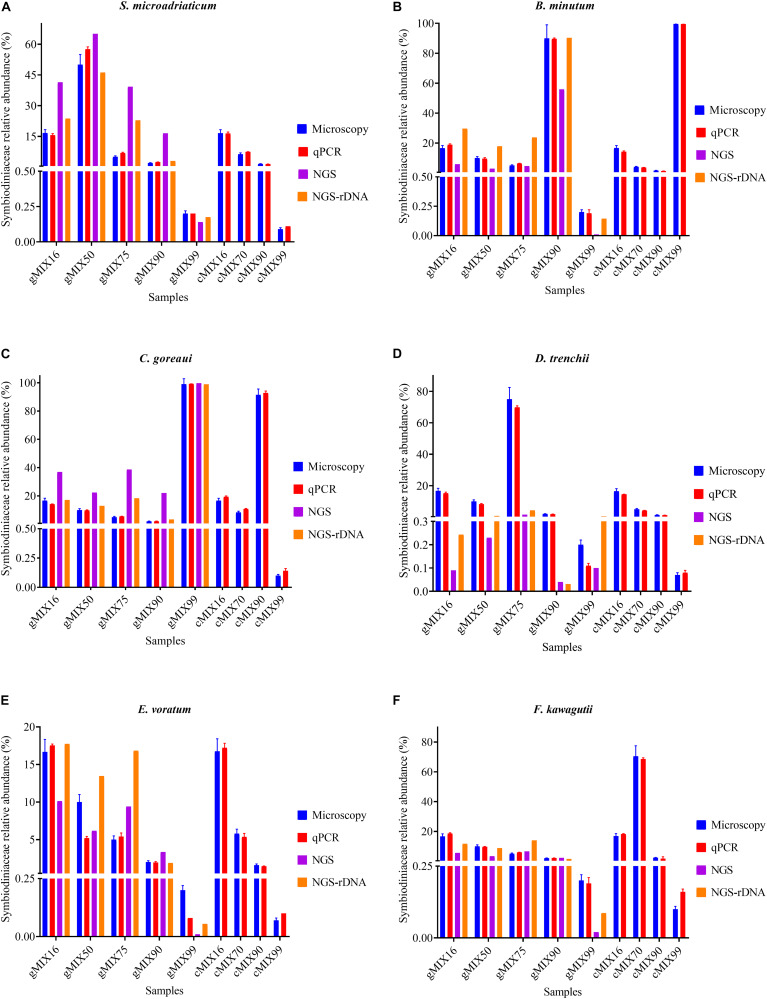
The Interrelationship between microscopic cell count, results of qPCR assay (ITS-STD) and Illumina HiSeq data without calibration (NGS) and with rDNA calibration (NGS-rDNA). Six Symbiodiniaceae species **(A–F)** were used to construct the nine artificially mixed samples (gMix16 –cMix99), In gMix set, genomic DNA from cultured species was mixed, with amount of each species corresponding to a certain cell number calculated based upon genome size data, while in cMix set, cells were combined based on microscopic count and DNA was extracted from the mixed samples (for more detailed information see Supplementary Table S1). Each plot summarizes the cell number ratio of one Symbiodiniaceae species in the mix. NGS was done only for the five gMix samples. Error bars represent SD of three replicates.

To assess the applicability of our protocol for natural coral holobiont samples, we conducted qPCR assays for mock communities. We mixed six cultured Symbiodiniaceae species (Supplementary Table S1) at different ratios, ranging from 0.07 to 99.58%, to examine how closely our qPCR assays can reconstruct a Symbiodiniaceae assemblage. Strong correlations coefficient was found between microscopic count and results of both qPCR tests (*r* > 0.99; *p* < 0.0001) and no significant changes were detected in cell number ratios between the microscopic count and both ITS-STD and Cell-STD qPCR assays (Wilcoxon signed-rank test, *p* > 0.05; Figure 5). For further comparison, Bland-Altman plots were used (Supplementary Figure S3), which revealed no significant bias between the microscopy and each of the two qPCR methods. This result on the one hand reinforced our genome size and ITS copy number data and on the other proved the effectiveness of primer sets for quantitatively detecting the target species even at low background levels within a Symbiodiniaceae assemblage.

### ITS Metabarcoding of Artificially Mixed Samples

**FIGURE 6:**
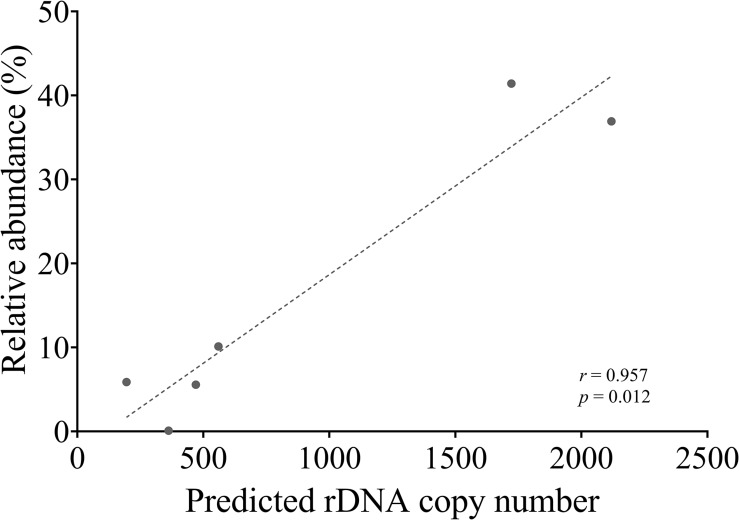
Correlation between NGS reads abundance (relative) and qPCR-estimated rDNA copy of each species in a mixed (gMix16) sample. In this sample, cell abundance of each species was equal.

To compare next-generation sequencing results with qPCR results in reflecting proportions of different species in the Symbiodiniaceae assemblages, NGS of ITS2 amplicon was conducted. Results showed in most cases NGS data deviated from microscopic and qPCR data, the latter two of which were very close to each other (Figure 5). The most conspicuous deviations occurred in the NGS data for the relative abundances of *C. goreaui* and *D. trenchii* across the various mixed samples with different species-to-species ratios. The NGS data overrepresented *C. goreaui*, corresponding to its high rDNA copy number, whereas *D. trenchii* was underrepresented, corresponding to its low rDNA copy number (Table 2). The smallest deviation was found in *F. kawagutii* and *E. voratum*, which had intermediate rDNA copy numbers (Table 2). Furthermore, the strong correlation between rDNA copy number and NGS data clearly indicated the strong influence of rDNA copy number on NGS data (Figure 6). After calibration with ITS copy numbers estimated in this study, a general improvement was observed (Figure 4). Only in rare cases calibrated results did not adequately eliminate the deviations from microscopic and qPCR results (*E. voratum* in gMIX75 sample) or instead created or increased the deviations (*D. trenchii* in gMIX75 and gMIX90 samples) (Figure 5).

**TABLE 2:** rDNA copy number of Symbiodiniaceae species estimated in previous and the present study.

Species	rDNA copy No./cell	Host/Source	References	Notes
*Breviolum* sp.	2 ± 1	*Aiptasia pallida*	Loram et al., 2007	qPCR (Bulk cell)
*Breviolum* sp.	7 ± 6	*Madracis mirabilis*	Loram et al., 2007	qPCR (Bulk cell)
*B. minutum*	116 ± 11.5	Database	Gong and Marchetti, 2019	Computational
*B. minutum*	195 ± 28	Culture (CCMP830)	This study	qPCR (Bulk cell)
*Cladocopium* sp.	984 ± 109 SE	*Acropora millepora* *A. tenuis* *Pocillopora damicornis*	Mieog et al., 2007	qPCR (Bulk cell)
*Cladocopium* sp.	500–22,000	*A. millepora* *A. tenuis* *P. damicornis*	Mieog et al., 2007	qPCR (Single cell)
*Cladocopium* sp. (C1)	C1 > A3,B1,D1a,E2 (C1 = 3–5X D1a)	Culture	Thornhill et al., 2007	qPCR (Bulk cell)
*C. goreaui*	2119 ± 217	Culture (CCMP2466)	This study	qPCR (Bulk cell)
*Durusdinium* sp.	3181 ± 69 SE	*A. millepora*	Mieog et al., 2007	qPCR (Bulk cell)
*Durusdinium* sp.	2300–12,000	*A. millepora*	Mieog et al., 2007	qPCR (Single cell)
*D. trenchii*	362 ± 23	Culture (CCMP3428)	This study	qPCR (Bulk cell)
*Effrenium voratum*	560 ± 65	Culture (CCMA192)	This study	qPCR (Bulk cell)
*Fugacium kawagutii*	160 ± 5.6	Database	Gong and Marchetti, 2019	Computational
*F. kawagutii*	470 ± 86	Culture (CCMP2468)	This study	qPCR (Bulk cell)
*Symbiodinium* sp.	17 ± 4	*Cassiopeia xamachana*	Loram et al., 2007	qPCR (Bulk cell)
*Symbiodinium* sp.	22 ± 6	*Bartholomea annulata*	Loram et al., 2007	qPCR (Bulk cell)
*S. microadriaticum*	1721 ± 125	Culture (CCMP828)	This study	qPCR (Bulk cell)

### Environmental Coral Samples

**FIGURE 7:**
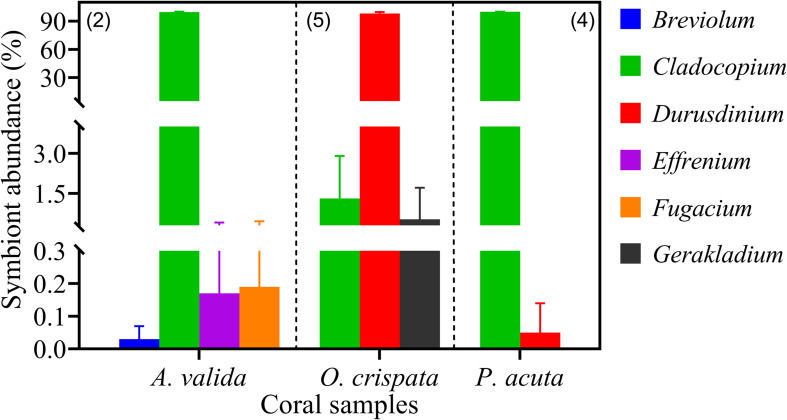
Symbiodiniaceae assemblages of three coral species from Hong Kong profiled using six Symbiodiniaceae genus-specific qPCR assays. Absolute quantification was achieved by applying cell number standard curve in qPCR. Number in brackets shows the number of sample replicates analyzed. Error bars indicate standard deviations among the replicates.

Three coral species from Hong Kong (HK) coral community, *Acropora valida, Oulastrea crispata*, and *Platygyra acuta*, were used to verify the reliability of qPCR assays in detecting and quantifying the different Symbiodiniaceae species from environmental corals. Results showed that apart from *S. microadriaticum*, positive amplification was yielded for all other six genera we examined (previously clade B – G) (Figure 7). Those amplicons were identified to genus level and confirmed to be the primer-targeted genera by the results of cloning, sequencing and blast analysis against the NCBI database. Our results showed dominance of the symbiont communities by *Cladocopium* (> 99.5%) in both *A. valida* and in *P. acuta*, and *Durusdinium* (>98.16%) in *O. crispata* corals. Our results also showed low relative abundances for species in *Breviolum* (0.05%), *Effrenium* (0.34%), and *Fugacium* (0.38%) in *A. valida, Durusdinium* (0.22%) in *P. acuta*; and species in *Cladocopium* (0.17%) and *Gerakladium* (2.97%) in *O. crispata.* Among these, *Breviolum*, *Effrenium, Fugacium* and *Gerakladium* sp., are reported the first time in corals in HK. This indicates that all the primer sets are good candidates for fishing the rare Symbiodiniaceae species from natural corals.

## Discussion

In this study, we have established an experimental procedure for developing and validating reliable and robust qPCR assays for identifying and quantifying Symbiodiniaceae genera. Following the MIQE guideline for qPCR design and assays (Bustin et al., 2009; Bustin and Huggett, 2017), seven Symbiodiniaceae genus-specific ITS2 primers were designed and verified computationally and experimentally using various types of samples. Compared with previously reported protocols, several quality assurance steps were added, including (1) aggressive cell disruption and microscopic check to ensure thorough cell lysis, (2) investigating cell loss during sampling and DNA loss during the extraction and purification process, and (3) using multi-approaches to validate the protocol. These steps are critical to achieve defined DNA recovery rate, accurate estimation of genome size and ITS copy number, and hence reliable qPCR results. Our protocol takes into consideration not only DNA loss in the purification step, as done in several previous studies (Mieog et al., 2009; Yamashita et al., 2011; Rouzé et al., 2017), but also losses due to cell loss and incomplete cell lysis in the preceding steps. The protocols will be useful for studies on these representative genera covered in this study. Because the primers were designed on the regions conserved within genus, these primers are potentially applicable to most species in respective genera, although verification using many more species is required. Nevertheless, the experimental procedure may serve as a template for similar attempts to develop new qPCR assays for other genera or species in the future.

### Importance to Determine Cell Loss and DNA Extraction Efficiency

Achieving an accurate absolute qPCR quantification is often compromised by poor DNA isolation procedure, unspecific primer set (Zinger et al., 2019) and the gene marker copy number variation across different species (Cunning and Baker, 2014). Consequently, successful and useful qPCR assay start with a good quality DNA isolated with a defined efficiency. Our CTAB-Zymo column method has been proven to be effective in achieving good quality DNA (Yuan et al., 2015). High DNA extraction efficiency, determined by degree of cell recovery during sample collection, completeness of cell lysis during incubation and homogenization, and DNA recovery rate during the DNA purification stage. High cell recovery, thorough cell lysis, and high efficiency of DNA recovery are crucial for detecting low-abundance species. Unaccounted loss in any of the steps causes errors in quantitative analyses, particularly in estimation of genome size and rDNA copy number per cell. Thus, the major first step in our protocol was to carefully investigate these losses and develop methods to calibrate for the losses. To account for cell loss, we examined the supernatant and the tubes before they were discarded after centrifugation to harvest cells and subtract the cell count from initial cell count from culture samples. Some cells might stick to the walls of the centrifuge tubes; therefore, before microscopic examination of the supernatant, we vortex-mixed tubes to detach those cells. We also used microscopic checking of the cell lysate during sample homogenization (bead-beating) to ensure thorough cell breakage in each sample. This is important because some Symbiodiniaceae assemblage in the corals may contain recalcitrant species that are hard to break. From our experience, it takes a substantial effort to break *F. kawagutii* cells completely.

Calibrating DNA yields for DNA recovery efficiency is necessary to obtain accurate estimates of cell genome size and rDNA copy number. The efficiency can be estimated from the slope of the linear regression of final DNA yield versus initial DNA, as we obtained from the control run of extracted DNA through the DNA purification procedure. The strong coefficient correlation in our study suggests that our DNA isolation process is robust and reliable over a dynamic range of DNA concentration, consistently producing a DNA recovery rate of about 71% (Figure 2B). With this defined DNA recovery efficiency, we were able to reconstruct the true DNA content per cell from measured DNA (Figure 2C). The Symbiodiniaceae genome sizes thus estimated are within the previously published range (1–5 Gbp) and close to reported flow cytometric values (Lajeunesse et al., 2005). Our estimated genome size of *F. kawagutii* is similar to both K-mer based estimations by Lin et al. (2015) and Liu et al. (2018). However, *C. goreaui* genome size estimated in the present and an earlier study (Lajeunesse et al., 2005) was twice greater than an earlier K-mer based estimate (Liu et al., 2018). It is possible that the genome of *C. goreaui* is more complex, hence challenging K-mer analysis, or alternatively, different strains of this species may possess drastically different sizes of genomes.

### rDNA Copy Number Variability

The higher copy number of rDNA confers higher detection sensitivity of this gene marker, this advantage comes with a shortcoming stemming from the cross-species copy number variations of this gene (Mieog et al., 2007; Thornhill et al., 2007; Correa et al., 2009; Gong and Marchetti, 2019). This shortcoming poses formidable challenges to efforts of quantifying the abundances of different Symbiodiniaceae species based on qPCR or metabarcoding methods (Herrera et al., 2009; Arif et al., 2014; Meistertzheim et al., 2019). To overcome the problem, estimating rDNA copy number for known species is essential. Using our protocol, we found that rDNA copy number for the species examined in this study ranged from 195 ± 28.97 to 2119 ± 217.17, more than 10-fold variation. Previously reported copy number values of *Symbiodinium* sp., *Breviolum* sp. (Loram et al., 2007), *B. minutum* and *F. kawagutii* (Gong and Marchetti, 2019) were lower than our estimates, but the overall trend among those species were comparable to our results (Table 2). In contrast to the copy number reported in Mieog et al. (2007), the estimated rDNA copy number ratio between *C. goreaui* and *D. trenchii* in Thornhill et al. (2007) was similar to our data. Although the divergences in rDNA copy number measurement can be attributed to species/strain type, the protocols used in DNA and qPCR processing may well be a source of the discrepancies (Gong and Marchetti, 2019). From biological and ecological perspectives, the widespread of *C. goreaui* and *S. microadriaticum* (Pochon et al., 2001; Coffroth and Santos, 2005; Cunning et al., 2015; Decelle et al., 2018) could be attributed to their higher rDNA gene copy number as a competitive trait in energy production and cell development (Rubio-Piña et al., 2016). However, from an analytical viewpoint, abundances of species with higher rDNA copy numbers in natural assemblages are expected to be overestimated. For instance, ITS copy number in *C. goreaui* is 10-fold higher than that in *B. minutum*, and failure to calibrate for the difference would substantially overestimate cell abundance of *C. goreaui* when in NGS assays for an assemblage in which both species coexist (Figure 5). Further work should expand taxon coverage in estimating rDNA copy number toward the goal of establishing a genome size and rDNA copy number database.

Our data also show that in ITS2 metabarcoding (NGS sequencing), calibrating with rDNA copy number resulted in significant improvement in profiling the Symbiodiniaceae community. The Illumina HiSeq result without calibration showed variable degrees of deviations in Symbiodiniaceae species relative abundances from microscopic count and qPCR assays, the latter two of which were very close to each other (Figure 5). Our results (Figure 6) revealed a strong correlation between Symbiodiniaceae species rDNA copy number and their relative abundances generated from Illumina HiSeq (*r* = 0.96; *p* < 0.01), which is evidence that rDNA copy number variations is the main influencing factor for incorrect relative species abundance information inferred from NGS analysis (Farrelly et al., 1995). The deviations were significantly reduced in most cases when the rDNA copy number was used to calibrate the relative abundance of each genus. This is consistent with recent efforts (Perisin et al., 2016; Gong and Marchetti, 2019). Systematic calibration for metabarcoding of natural coral samples in the future calls for a concerted research effort to develop a comprehensive rDNA copy number database. We also noticed that besides rDNA copy number, some other factors might influence NGS profiling of a Symbiodiniaceae community, as in a small number of cases, calibration using rDNA copy number did not fully eliminate the deviations from microscopic and qPCR results (e.g., *D. trenchii* in gMIX75 and gMIX90 samples) or even increased the deviations (e.g., *E. voratum* in gMIX75 sample). These other influencing factors might include biases in sequencing and bioinformatics processing (Thornhill et al., 2013; Hume et al., 2018).

### Multi-Approach to Verify qPCR Specificity

Use of *in silico* and experimental analyses is helpful in verifying PCR specificity. For experimental analysis, inclusion of pure species and mock community samples allows examination if co-existence of multiple non-target species might interfere with quantification of target species, as in natural coral samples. For *in silico* analysis, NCBI free online primer tools and other software such as MFEprimer2 with an appropriate database (Qu et al., 2009; Qu and Zhang, 2015; Bustin and Huggett, 2017) are helpful. The specificity of the resulting primers relies on the completeness of the database used in the *in silico* analysis. As such, primers may need to be reevaluated over time against the rapidly growing database (Bustin and Huggett, 2017).

### *In vitro* qPCR Verification

In the experimental (*in vitro*) validation of a qPCR assay, melting curve analysis and sequencing of the amplicon are commonly used tools for ensuring specificity, and PCR efficiency from the standard curve is an indicator of optimality of PCR conditions. In our study, the single melting temperature (Tm) ≥ 78°C is a qPCR indicator of primer pair specificity and absence of primer dimers (Bustin and Huggett, 2017). The strong linear correlation (*R*^2^ ≥ 0.999, *p* < 0.0001) along with high efficiency (Table 1), generated by plotting the log (ITS copy number) or log (cell number) versus Cq values (Supplementary Figure S2), demonstrates the reliability of the qPCR conditions. Moreover, the similar Cq values between target amplicon within a mixed sample and pure species sample (Figure 3) indicate that the presence of closely related sequences did not interfere with the qPCR reactions under the conditions we used. Comparing the microscopic cell counts data with both qPCR (Cell-STD and ITS-STD) assays, across nine different artificially mixed samples and six Symbiodiniaceae genus-specific primer sets, generates strongly matched results (Figure 5). This result demonstrates that the qPCR assay is very sensitive and specific in detecting background Symbiodiniaceae species below 0.1%, equivalent to 1 cell of the targeted species coexisting with more than 1000 cells of closely related taxa. Whether we defined the sensitivity by the (2^^*Cq(Z*)–32^) formula, where (Z) is the Cq values of the dominant symbiont and 32 is the cutoff cycle (Correa et al., 2009), or by the lowest amount of the nucleic acid that can be detected (Rouzé et al., 2017), our qPCR assays (values) exhibit a comparable or higher sensitivity (0.001% relative abundance or 100 rDNA copy) than the previous reports (Correa et al., 2009; Yamashita et al., 2011; Lin et al., 2017; Rouzé et al., 2017).

Furthermore, the Blant–Altman analysis provides another way of validating qPCR assays. The results in the present study showed good agreement between each of the qPCR assays and the microscopic cell count, as the discrepancy detected between their respective averages was always close to 0.0 and the majority of the data fell within the 95% confidence intervals range. This outcome is clear evidence that primer sets and qPCR assays designed in this study are applicable for quantifying Symbiodiniaceae species in mixed assemblages.

### Applicability of qPCR for Natural Coral Samples

Useful qPCR assays should be applicable to natural coral samples. Our initial tests indicate that the protocol developed here, following the procedure established in this study, is applicable to natural coral samples. From *A. valida, O. crispata*, and *P. acuta* at HK marginal coral communities, we detected six Symbiodiniaceae ITS2 genera (Figure 7), four of which is documented for the first time in these coral species in this geographic location. Although *Breviolum*, *Fugacium*, and *Gerakladium* had been well documented as coral symbionts in various other locations (Pochon et al., 2014), *E. voratum* associated with *A. valida* is unexpected because this species has not been reported as a symbiont of corals. It needs to be noted our testing was done on a small number of natural coral samples only, and the qPCR assays need to be further examined on a wider range of corals from different locations. Furthermore, additional coral washing steps are warranted in future studies to exclude any possibility of contamination by cells attached to the surface of the coral samples. *E. voratum* has once been reported in association with *Alveopora japonica* from marine water nearby our study site, another coral from the family Acroporidae, but it was suspected to be an epibiont on the surface of the coral (Jeong et al., 2014). Similar suspicion could be raised for *Gerakladium* detected in *O. crispata* in the present study, because this genus has mostly been documented as symbionts of foraminifera and sponges (Pochon and Gates, 2010). Special attention should be given in future studies to thorough cleaning of coral surface after sample collection to avoid contamination. However, *Gerakladium* has also been reported from corals in the Great Barrier Reef (Van Oppen et al., 2005), Hawaii (Stat et al., 2013), the geographical extremes of western Australia (Thomas et al., 2014), and the Sea of Oman (Ziegler et al., 2017). In addition, our estimate of the contribution of *Cladocopium* to the symbiont assemblage in *O. crispata* was lower than that in a previous report (Ng and Ang, 2016), likely due to higher rDNA copy number in *Cladocopium*, as found for the cultured strain in the present study. We found that the partial ITS2 sequences of the wild Symbiodiniaceae species, except *Durusdinium* sp. in *O. crispata*, were identical to our cultured strains of the corresponding species. It is thus possible, but remaining to be verified, that the rDNA copy numbers of the wild and cultured strains might be comparable. To verify this, future research can use single-cell PCR on cells isolated directly from coral samples collected *in situ*.

## Data Availability Statement

Raw sequence data were deposited in the NCBI as follows: Illumina raw read data in Sequence Read Archive (SRA) under accession no. PRJNA599937 and the clone sequences under the accession nos. MN917869–MN917983. All other data are included in the Supplementary Material.

## Author Contributions

SL conceived and supervised the project. SL, OS, LL, and XL designed the study. TN and PA collected and processed the environmental coral samples. OS conducted the experiments, data analysis and wrote the first draft of the manuscript. All authors edited and approved the manuscript.

## Conflict of Interest

The authors declare that the research was conducted in the absence of any commercial or financial relationships that could be construed as a potential conflict of interest.

## Footnotes

1 https://www.ncbi.nlm.nih.gov/tools/primer-blast/

2 http://biotools.nubic.northwestern.edu/OligoCalc.html
