# Supplementary material for: Genome Size, rDNA Copy, and qPCR Assays for Symbiodiniaceae
Source: Front Microbiol. 2020 May 26;11:847. doi: 10.3389/fmicb.2020.00847 (PMC7264167; doi:10.3389/fmicb.2020.00847)
Supplement: Supplementary file 2 [file Image_2.pdf]

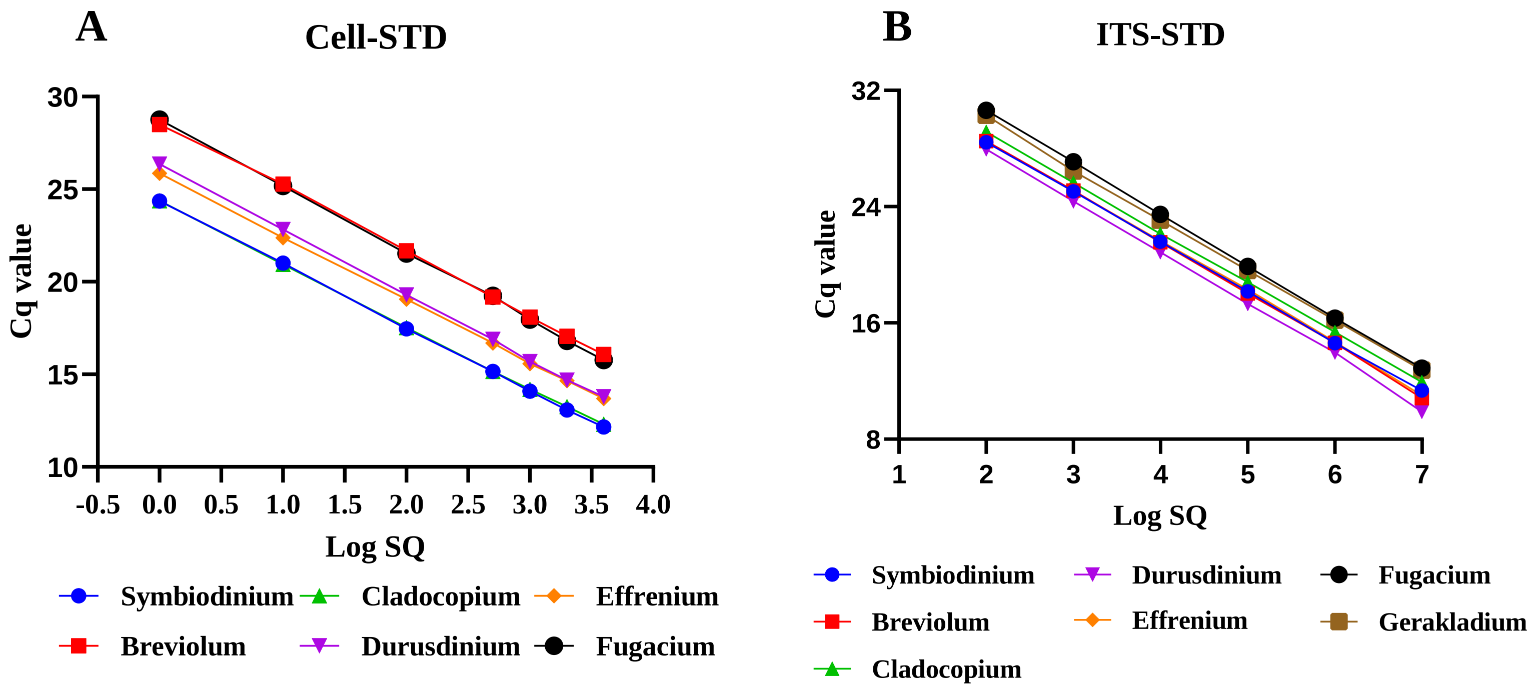

Supplementary Figure S2. Symbiodiniaceae genus-specific primer set standard curves. Tow standards were generated, (A) cell number based standard (Cell-STD): constructed as 1, 10, 100, 500, 1000, 2000 and 4000 cell per qPCR reaction. (B) ITS copy number based standard (ITS-STD) constructed from a purified ITS gene as serial of 10-fold dilutions 10^2 to 10^7 copy number per qPCR reaction. Cq values plotted against (A) Log (Cell number) and (B) Log (ITS copy number). Standard deviation of two plates replicate denoted as error bars.
