# Supplementary material for: Genome Size, rDNA Copy, and qPCR Assays for Symbiodiniaceae
Source: Front Microbiol. 2020 May 26;11:847. doi: 10.3389/fmicb.2020.00847 (PMC7264167; doi:10.3389/fmicb.2020.00847)
Supplement: Supplementary file 3 [file Image_3.pdf]

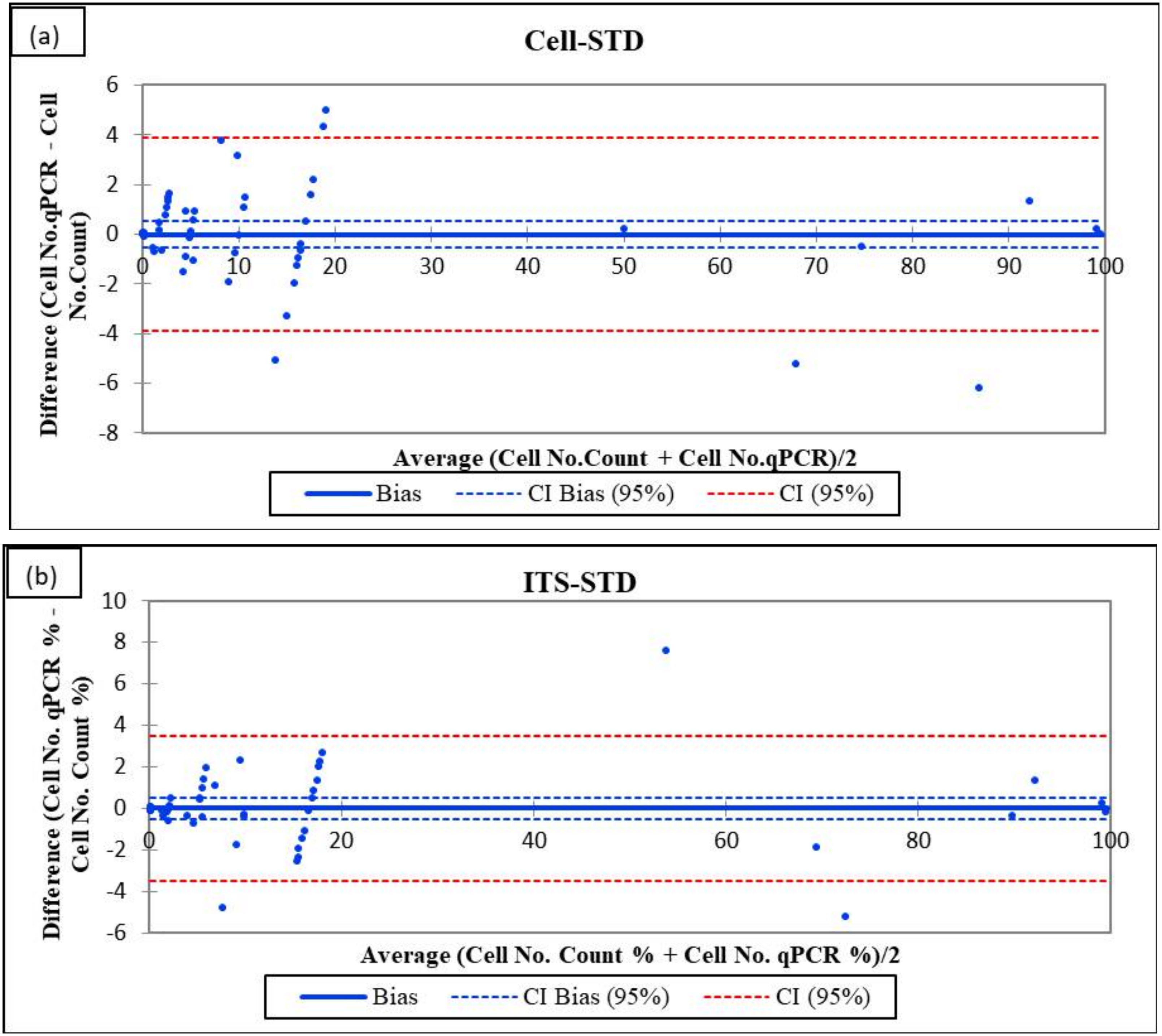

Supplementary Figure S3. Assessment of agreement level between light microscopy and qPCR assays using Bland-Altman plots. (a) Agreement between light microscopy and Cell-STD qPCR assays. Mean bias 0.00% ± 1.99%, 95% confidence intervals range -3.90 to 3.90%. (b) Agreement between light microscopy and ITS-STD qPCR assays. Mean bias - 0.003% ± 1.80%, 95% confidence intervals range -3.52% to 3.52%. Nine different samples were examined using six Symbiodiniaceae genus-specific primer sets.
