# Supplementary material for: Genome Size, rDNA Copy, and qPCR Assays for Symbiodiniaceae
Source: Front Microbiol. 2020 May 26;11:847. doi: 10.3389/fmicb.2020.00847 (PMC7264167; doi:10.3389/fmicb.2020.00847)
Supplement: Supplementary file 4 [file Table_1.docx]

**Supplementary Table S1.** Symbiodiniaceae species composition used to construct nine artificial mixed samples. The cell number percentage (%) of six different cultured Symbiodiniaceae species used to construct two sets of artificial mixed samples; (cMix) based on real cell counting number and (gMix) based on genomic DNA transformed as cell number depending upon the genome size values generated in this study (see results).

| **Sample type** | **Sample ID** | ***S. microadriaticum*** | ***B. minutum*** | ***C. goreaui*** | ***D. trenchii*** | ***E. voratum*** | ***F. kawagutii*** |  |
| --- | --- | --- | --- | --- | --- | --- | --- | --- |
| **cMix** | cMix16 | 16.64 | 16.71 | 16.63 | 16.42 | 16.73 | 16.85 |  |
|  | cMix70 | 6.35 | 4.09 | 8.35 | 4.97 | 5.80 | 70.45 |  |
|  | cMix90 | 1.57 | 1.68 | 91.49 | 1.36 | 1.59 | 2.32 |  |
|  | cMix99 | 0.09 | 99.58 | 0.10 | 0.07 | 0.07 | 0.10 |  |
| **gMix** | gMix16 | 16.67 | 16.67 | 16.67 | 16.67 | 16.67 | 16.67 |  |
|  | gMix50 | 50.00 | 10.00 | 10.00 | 10.00 | 10.00 | 10.00 |  |
|  | gMix75 | 5.00 | 5.00 | 5.00 | 75.00 | 5.00 | 5.00 |  |
|  | gMix90 | 2.00 | 90.00 | 2.00 | 2.00 | 2.00 | 2.00 |  |
|  | gMix99 | 0.20 | 0.20 | 99.00 | 0.20 | 0.20 | 0.20 |  |
