# Supplementary material for: Genome Size, rDNA Copy, and qPCR Assays for Symbiodiniaceae
Source: Front Microbiol. 2020 May 26;11:847. doi: 10.3389/fmicb.2020.00847 (PMC7264167; doi:10.3389/fmicb.2020.00847)
Supplement: Supplementary file 5 [file Table_2.docx]

**Supplementary Table S2.** qPCR specificity assays of Symbiodiniaceae genus-specific primer sets. Each genus-specific primer sets were run against all Symbiodiniaceae species (target and non-target amplicon). Duplicate 1 ng DNA/reaction were used as template except for *Gerakladium* sp*.* where ITS2 purified PCR product was used instead*.* Cq values reported as an average of duplicate.

| DNA sample | Symbiodiniaceae genera –specific qPCR Primer | | | | | | | |
| --- | --- | --- | --- | --- | --- | --- | --- | --- |
|  | *Symbiodinium* | *Breviolum* | | *Cladocopium* | *Durusdinium* | *Effrenium* | *Fugacium* | *Gerakladium* |
| *S. microadriaticum* | 15.32 | 34.44* | None | | None | None | None | 33.28* |
| *B. minutum* | None | 17.31 | None | | None | None | None | None |
| *C. goreaui* | None | 34.42* | 15.50 | | None | None | None | None |
| *D. trenchii* | None | None | None | | 18.38 | None | None | None |
| *E. voratum* | None | None | None | | None | 15.93 | None | None |
| *F. kawagutii* | None | None | None | | None | None | 18.13 | None |
| *Gerakladium* sp. | None | None | None | | None | None | None | 11.18 |

None = No amplicon. * = Cq value of non-target amplicon
