# Supplementary material for: Genome Size, rDNA Copy, and qPCR Assays for Symbiodiniaceae
Source: Front Microbiol. 2020 May 26;11:847. doi: 10.3389/fmicb.2020.00847 (PMC7264167; doi:10.3389/fmicb.2020.00847)
Supplement: Supplementary file 6 [file Table_3.docx]

**Supplementary Table S3.** BLAST search re-identified the misannotated Symbiodiniaceae sequences. The confused species names and the real species names obtained from the BLAST search.

| **Misannotation** | **Accession No.** | **BLAST search** |
| --- | --- | --- |
| *Gymnodinium linucheae* | AF333509 | *Symbiodinium* type A |
| *Symbiodinium kawagutii* | AF180121 | *Symbiodinium t*ype A |
| *Dinophyceae sp* | MH974805 | *Symbiodinium* type B |
| *Symbiodinium* sp. B1 | KP761367 | *Symbiodinium* type C |
| *Symbiodinium* sp. A1 | KP761365 | *Symbiodinium* type C |
| *Symbiodinium* sp. type A | KM041029 | *Symbiodinium* type C |
| *Gambierdiscus excentricus* | KX395824 | *Symbiodinium* type D |
| *Symbiodinium minutum* | LC002802 | *Symbiodinium* type D |
| *Symbiodinium* sp. G15 | AY160123 | *Symbiodinium* type E |
| *Symbiodinium* sp. ZX11 | GU362428 | *Symbiodinium* type E |
| *Symbiodinium* sp. clade_C | AF360576 | *Symbiodinium* type C/F |
| *Symbiodinium* sp. CCMP2455 | LK934673 | *Symbiodinium* type F |
| *Symbiodinium* sp. C3 | AF333516 | *Symbiodinium* type C3/F |
| *Symbiodinium* sp. PtBr/clade C3 | HQ317747 | *Symbiodinium* type C3/F |
