# Supplementary material for: Genome Size, rDNA Copy, and qPCR Assays for Symbiodiniaceae
Source: Front Microbiol. 2020 May 26;11:847. doi: 10.3389/fmicb.2020.00847 (PMC7264167; doi:10.3389/fmicb.2020.00847)
Supplement: Supplementary file 7 [file Table_4.docx]

**Supplementary Table S4.** qPCR consistency test of Symbiodiniaceae genus-specific primer sets. Consistency of seven Symbiodiniaceae genus-specific primer sets were expressed as ΔCq over eight different annealing temperature. qPCR temperature gradient analysis assays were run using 1 ng DNA for each species except *Gerakladium* where PCR product were used. Cq values reported as an average of duplicate.

| Temperature (°C) | *Symbiodinium* | *Breviolum* | *Cladocopium* | *Durusdinium* | *Effrenium* | *Fugacium* | *Gerakladium* |
| --- | --- | --- | --- | --- | --- | --- | --- |
| **64.0** | 12.18 | 13.33 | 13.01 | 11.35 | 12.20 | 13.19 | 13.32 |
| **63.6** | 12.21 | 13.13 | 13.05 | 11.26 | 12.18 | 13.19 | 13.18 |
| **62.7** | 12.20 | 12.78 | 13.03 | 11.12 | 12.13 | 13.18 | 13.09 |
| **62.1** | 12.18 | 12.72 | 13.07 | 11.12 | 11.94 | 13.31 | 12.96 |
| **59.2** | 12.27 | 12.94 | 13.03 | 11.33 | 12.05 | 13.36 | 12.90 |
| **57.6** | 12.26 | 13.45 | 13.06 | 11.19 | 12.14 | 13.51 | 13.01 |
| **56.5** | 12.35 | 14.02 | 12.99 | 11.42 | 12.32 | 13.73 | 13.10 |
| **56.0** | 12.27 | 14.23 | 12.99 | 11.36 | 12.21 | 13.90 | 13.07 |
| **Range (ΔCq)** | **0.17** | **1.51** | **0.08** | **0.30** | **0.38** | **0.72** | **0.42** |
