# Supplementary material for: Genome Size, rDNA Copy, and qPCR Assays for Symbiodiniaceae
Source: Front Microbiol. 2020 May 26;11:847. doi: 10.3389/fmicb.2020.00847 (PMC7264167; doi:10.3389/fmicb.2020.00847)
Supplement: Supplementary file 1 [file Image_1.pdf]

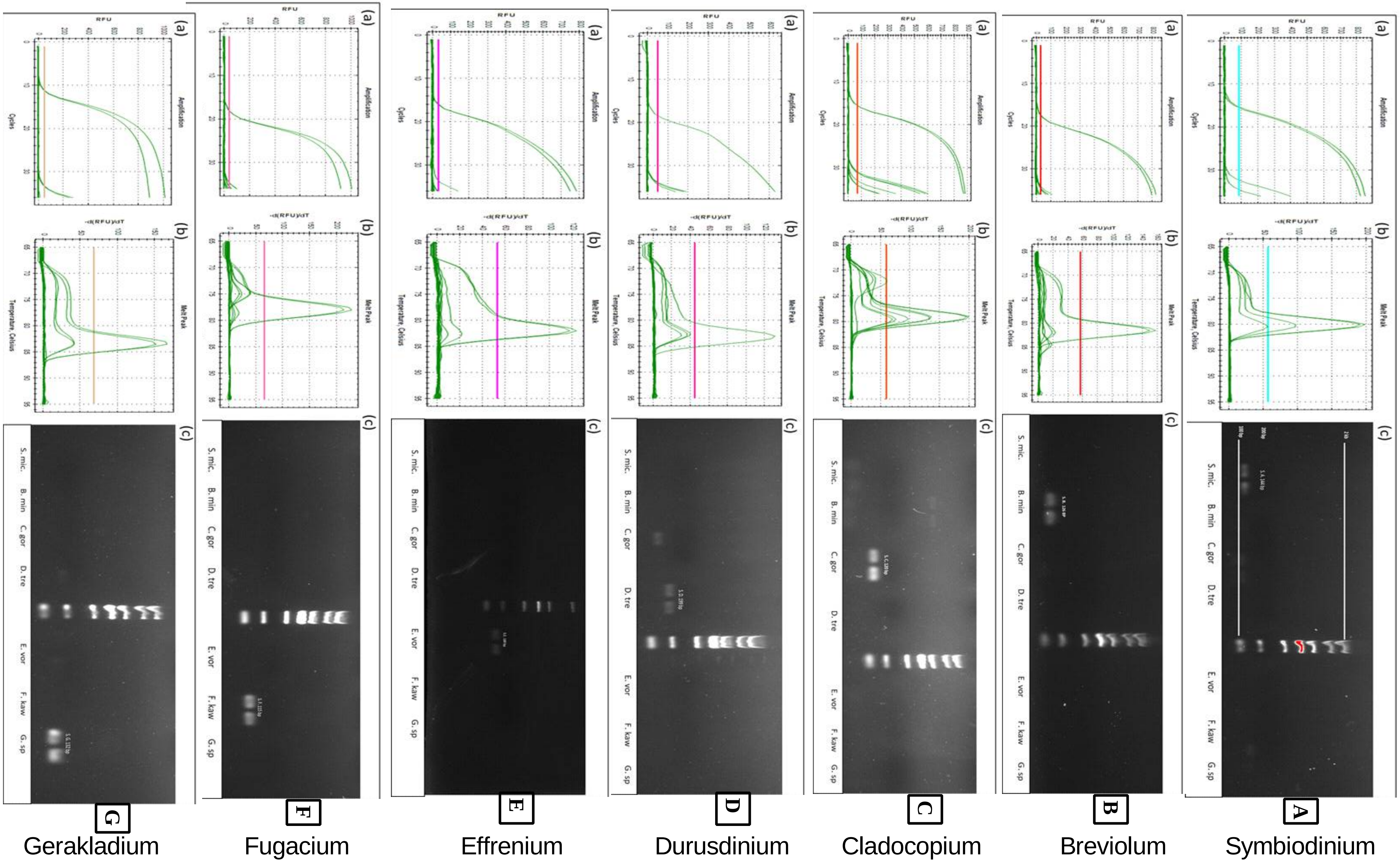

Supplementary Figure S1. Symbiodiniaceae genera primer set specificity test. Each Symbiodiniaceae genus (Symbiodinium, Breviolum, Cladocopium, Durusdinium, Effrenium, Fugacium and Gerakladium) specific primer set were run against all other Symbiodiniaceae genera. At the end of each qPCR assays positive (target) and six negative (non-target) amplicons were imagined on Agarose gel (1%) under UV light. All assays were run in 12 µl total volume and 1 ng DNA per qPCR reaction. Purified ITS2 PCR product was used as a template for Gerakladium sp. (a) qPCR amplicon. (b) qPCR melting temperature. (c) Gel image.
